# Evaluation of Different Regimens of Palliative Radiation Therapy for Symptomatic Bone Metastases: An Audit From a Tertiary Care Hospital in Jharkhand, India

**DOI:** 10.7759/cureus.53622

**Published:** 2024-02-05

**Authors:** Suhail Ahmed, Aaditya Prakash, Amitabh Kumar Upadhyay

**Affiliations:** 1 Radiation Oncology, Meherbai Tata Memorial Hospital, Jamshedpur, IND; 2 Radiation Oncology, Tata Main Hospital, Jamshedpur, IND; 3 Medical Oncology, Tata Main Hospital, Jamshedpur, IND

**Keywords:** bone metastasis, cancer, single fraction, radiotherapy, pain

## Abstract

Background

This study aimed to assess the efficacy of different radiation therapy regimens in treating patients with symptomatic bone metastases.

Methodology

A retrospective study was conducted by assigning patients with symptomatic bone metastases from different primary cancers into three groups, namely, Arms A, B, and C. The radiation dose delivered in each arm was as follows: 8 Gray (Gy) in a single fraction for Arm A, 20 Gy in five fractions at the rate of 4 Gy per fraction for Arm B, and 30 Gy in 10 fractions at the rate of 3 Gy per fraction for Arm C. Each arm consisted of 15 patients. A comparison was conducted across all three arms to evaluate pain relief based on the Visual Analog Scale (VAS), performance score improvement based on the Eastern Cooperative Oncology Group (ECOG), and analgesic requirement based on the World Health Organization (WHO) step ladder at one week, one month, and three months.

Results

The pain relief was measured using the VAS in three different arms, i.e., Arm A, B, and C. After one week, the pain relief was 66.67%, 60%, and 60%, respectively. After one month, it was 73.33% in all three arms. At three months, it was 80%, 86.67%, and 86.67%, respectively. The study also measured the improvement in the ECOG performance score. The improvement in all three arms was 60% after one week and 66.67% in Arm A and 73.33% in Arms B and C after one month. After three months, the improvement was 73.33%, 80%, and 80% in Arms A, B, and C, respectively. The decrease in analgesic usage was also measured in all three arms. After one week, it was 60% in all three arms. After one month, it was 66.67%, 73.33%, and 73.33% in Arms A, B, and C, respectively. At three months, it was 73.33%, 80%, and 80% in Arms A, B, and C, respectively. No significant statistical difference was found between the three arms.

Conclusions

The efficacy of a single 8 Gy arm was almost equivalent to that of other arms of multifractionated regimens in terms of improvement in pain and performance score and decreased use of analgesics for a short duration of follow-up. For high-volume cancer centers and patients with economic constraints, a single-fraction regime provides effective palliation for painful bone metastases.

## Introduction

In India, lung and breast cancers are responsible for 70% of cases of bone metastases [1]. Additionally, 85% of the cases that either present or die due to bone metastases are associated with lung, breast, or prostate cancers [2,3]. Other less common types of bone metastases include malignant melanoma, renal cell carcinoma, thyroid cancer, and gastrointestinal malignancies. Multiple myeloma and lymphomas can also lead to bone destruction, causing pain. Bone metastases are a frequent occurrence in cancer patients and are the third most common site after lung and liver metastasis [4]. It is estimated that between 40% and 70% of individuals with primary lung, breast, and prostate cancers will develop skeletal metastases at some point in their life [5]. Patients with bone metastases due to breast and prostate cancer typically have a median overall survival of two to four years, while those with lung cancer have an overall survival of six months [5-7]. Bone metastases are most commonly found in the axial skeleton, which includes the spine, pelvis, and ribs [8]. The lumbar spine is the most frequently affected site in the spine, while the proximal femur and humerus are most commonly affected in the appendicular skeleton [8]. These metastases are often classified as osteolytic, osteoblastic, or mixed [9].

The management of bone metastases involves several treatment options, including radiotherapy, chemotherapy, targeted therapy with anti-RANKL antibody denosumab, hormone therapy, surgery, radionuclide, and supportive therapy. Depending on the patient’s condition, these treatments can be administered individually or in combination [10]. Patients require palliative treatment to alleviate symptoms such as pain, difficulty with ambulation and movement, pathological fractures, neurological deficits, spinal cord compression, and other related issues. Palliative radiation therapy has been found to enhance the quality of life of patients suffering from advanced carcinoma despite the poor survival rate in such cases. According to studies, around 75% of patients report feeling relieved from pain for three to six months after undergoing the therapy [11]. This study aims to assess the effectiveness of the most appropriate radiation therapy plan for treating symptomatic bone metastases, taking into account several factors such as pain relief, performance score, and analgesic use. Many cancer centers in India treat a large number of patients with skeletal metastases. These patients often come from poor economic backgrounds. This study aims to determine the most effective radiotherapy treatment for symptomatic bone metastases in a resource-constrained country.

## Materials and methods

**Figure 1:**
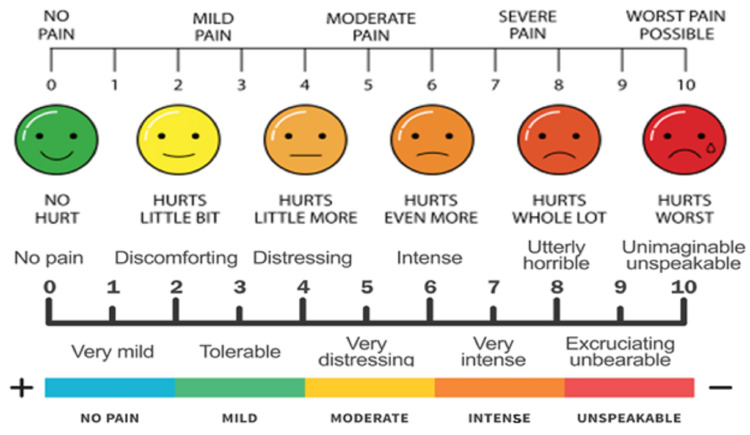
Visual Analog Scale.

This retrospective study was conducted in the Department of Radiation Oncology, Meherbai Tata Memorial Hospital Jamshedpur, India, and included patients treated between March 2022 and November 2022. This study underwent an internal review by the board. The study included 45 patients who had symptomatic bone metastases from any primary source (Table 1). Re-irradiation to the same site and poor general condition patients with multiple comorbidities were excluded. Patients who met the inclusion criteria were recruited into the study, and informed consent was obtained. Fifteen patients were included in the following three groups: Arm A received 8 Gy in a single fraction, Arm B received 20 Gy in five fractions, and Arm C received 30 Gy in 10 fractions (Table 2). Patients were allocated and evaluated for pain relief based on the Visual Analog Scale (VAS) (Figure 1), performance score improvement based on the Eastern Cooperative Oncology Group (ECOG), and analgesic requirements based on the World Health Organization (WHO) step ladder one week, one month, and three months after treatment. Table 1 and Table 2 present the patient characteristics in an organized manner. Of the 45 patients included in the study, 27 (60%) had metastases to the spine, 14 (31.11%) to the pelvis, two (4.44%) to the ribs, and one (2.22%) each to the sternum and femur.

**Table 1:** Primary tumor or tumor causing bone destruction.

Primary tumor/Tumor causing bone destruction	Number of patients
Lung cancer	17 (37.8%)
Breast cancer	14 (31%)
Prostate cancer	9 (20%)
Cervical cancer	2 (4.44%)
Multiple myeloma	3 (6.67%)

**Table 2:** Age distribution of patients in the three treatment arms. Arm A = 8 Gy in a single fraction; Arm B = 20 Gy in five fractions (4 Gy/fraction); Arm C = 30 Gy in 10 fractions (3 Gy/fraction)

Age (years)	Arm A (n = 15)	Arm B (n = 15)	Arm C (n = 15)	Total)
<30	0	0	0	0
30–39	0	2	0	2
40–49	3	3	2	8
50–59	5	3	5	13
60–69	4	4	6	14
>70	3	3	2	8

Patient evaluation criteria

Pain score based on VAS (Table 3), ECOG performance scale (Table 4), and analgesic requirement based on the WHO step ladder (Table 5) [12] for cancer pain management were the evaluation criteria for this study, which was recorded at the baseline (Tables 6-8), one week, one month, and three months after completion of treatment. The VAS [13] evaluated pain score assessment and relief. Pain relief was defined as a decrease in pain score by at least two points concerning the pretreatment value based on VAS. Improvement in performance status was considered as an improvement in ECOG grade by at least one grade, but no deterioration concerning pretreatment value at any point during follow-up from 1 to 12 weeks after irradiation. A decrease in analgesic use was a shift toward at least one step on the lower side of the WHO step ladder for cancer pain management. Adjuvant medications such as steroids, anxiolytics, antidepressants, and anticonvulsants were added for the management of neuropathic pain.

**Table 3:** Visual Analog Scale for pain assessment.

Score	Severity of pain
0	No pain
1–3	Mild pain
4–6	Moderate pain
7–9	Very severe pain
10	Worst pain possible

**Table 4:** The Eastern Cooperative Oncology Group grading system of functional status.

Grade 0	Fully active, able to carry on all pre-disease performance without any restrictions
Grade 1	Restricted in physically strenuous activity but ambulatory and able to carry out work of a light or sedentary nature, e.g., light housework, office work
Grade 2	Ambulatory and capable of self-care but unable to carry out any work activities. Up and about more than 50% of waking hours
Grade 3	Capable of only limited self-care, confined to the bed or chair for more than 50% of waking hours
Grade 4	Completely disabled, cannot carry out any self-care, totally confined to the bed or chair
Grade 5	Dead

**Table 5:** WHO step ladder for the management of cancer pain. NSAIDs: non-steroidal anti-inflammatory drugs; WHO: World Health Organization

WHO step ladder	Drugs/Interventions
1	NSAIDS
2	NSAIDS + weak opioid
3	NSAIDS + strong opioid
4	Invasive techniques such as nerve block, neurolysis, or surgical and other interventions

**Table 6:** VAS at the baseline or the first visit before radiotherapy. VAS: Visual Analog Scale

	Number of patients	VAS score
Arm A	10	4–6
	5	7–9
Arm B	7	7–9
	8	4–6
Arm C	9	4–6
	6	7–9

**Table 7:** ECOG performance score at the baseline or the first visit before radiotherapy. ECOG: Eastern Cooperative Oncology Group

	Number of patients	ECOG score
Arm A	10	3
	5	4
Arm B	7	3
	8	2
Arm C	9	2
	6	3

**Table 8:** Analgesic usage at the baseline or first visit before radiotherapy.

	Number of patients	WHO step ladder number for analgesics
Arm A	10	2
	5	3
Arm B	7	2
	8	3
Arm C	9	1–2
	6	2

Follow-up

Patients were monitored at different stages, i.e., during radiation treatment, and at one week, one month, and three months post-treatment.

Positioning and technique

All patients were treated in a supine, reproducible position with adequate immobilization with a thermoplastic mask or vacuum lock. The target volumes were delineated on simulated CT scan images. The metastatic gross tumor volume was covered with 2-3 cm margins proximally, distally, and circumferentially to create the planned tumor volume (PTV) [14]. For spinal lesions, the field borders included two vertebral bodies above and below the diseased/painful vertebrae with the circumferential margin 5 mm beyond the tip of the transverse processes to create the PTV [14]. All patients were treated with anterior-posterior and posterior-anterior fields except for the sternal metastasis, which was treated with two parallel opposed fields. A Varian True beam with a 6 MV photon beam was used for treatment.

Statistical analysis

The study examined table comparisons utilizing the chi-square test. Vertical comparison was performed to evaluate the efficacy of multifraction radiation therapy among the three arms of treatment. The aforementioned arms were compared and interpreted at the end of one week, one month, and three months. The results were interpreted based on pain relief, enhancement in performance status, and decreased analgesics. Horizontal comparison was done to determine if the response to treatment was achieved or not. If the response was achieved, it was analyzed to ascertain the duration for which it persisted, irrespective of the radiation therapy regimen. The statistical analysis was performed employing SPSS version 11.5 (SPSS Inc., Chicago, IL, USA).

## Results

Response to treatment

The evaluation of pain relief based on VAS in Arms A, B, and C revealed the following results: at one week, Arms A, B, and C showed a 66.67%, 60%, and 60% improvement, respectively. At one month, all three arms exhibited a 73.33% improvement, and at three months, Arms A, B, and C demonstrated 80%, 86.67%, and 86.67% improvement, respectively. The improvement in ECOG performance score was 60% in all three arms at one week, followed by 66.67% (Arm A), 73.33% (Arm B), and 73.33% (Arm C) at one month. At three months, the improvement was 73.33% (Arm A), 80% (Arm B), and 80% (Arm C). The decrease in analgesic usage was 60% in all three arms at one week, followed by 66.67%, 73.33%, and 73.33% at one month, and 73.33%, 80%, and 80% at three months, respectively. No statistically significant difference was found between the various arms.

Response rates in terms of pain relief are depicted in Table 9. Adequate pain relief was achieved for most patients during follow-up till three months of post-radiation. Improvements in performance status are depicted in Table 10. A decrease in analgesic requirement based on the WHO step ladder is depicted in Table 11.

**Table 9:** Pain relief with treatment. Arm A = 8 Gy in a single fraction; Arm B = 20 Gy in 5 fractions (4 Gy/fraction); Arm C = 30 Gy in 10 fractions (3 Gy /fraction). VAS: Visual Analog Scale

Variable	At one week (response in percentage) (VAS)	At one month (response in percentage) (VAS)	At three months (response in percentage) (VAS)	P-value
Arm A	10/15 (66.67%) (4–6)	11/15 (73.33%) (4–6)	12/15 (80%) (1–3)	0.71
	5/15 (33.33%) (7–9)	4/15 (26.67%) (7–9)	3/15 (20%) (7–9)	
Arm B	9/15 (60%) (4–6)	11/15 (73.33%) (1–3)	13/15 (86.67%) (0–3)	0.26
	6/15(40%) (7–9)	4/15(26.67%) (7–9)	2/15(13.33%) (7–9)	
Arm C	9/15 (60%) (4–6)	11/15 (73.33%) (4–6)	13/15 (86.67%) (0–3)	0.26
	6/15 (40%) (7–9)	4/15 (26.67%) (7–9)	2/15 (13.33%) (7–9)	
P-value	0.91	1	0.84	-

**Table 10:** Improvement in performance status. Arm A = 8 Gy in a single fraction; Arm B = 20 Gy in five fractions (4 Gy/fraction); Arm C = 30 Gy in 10 fractions (3 Gy /fraction) ECOG: Eastern Cooperative Oncology Group; PS: performance score

Variable	At one week (response in percentage) (ECOG PS)	At one month (response in percentage) (ECOG PS)	At three months (response in percentage) (ECOG PS)	P-value
Arm A	9/15 (60%) (3)	10/15 (66.67%) (3)	11/15 (73.33%) (3)	0.74
	6/15 (40%) (3 in four patients and 4 in two patients)	5/15 (33.33%) (3 in 3 patients and 4 in two patients)	4/15 (26.67%) (3 in two patients and 4 in two patients)	
Arm B	9/15 (60%) (3)	11/15 (73.33%) (3)	12/15 (80%) (2)	0.47
	6/15 (40%) (3)	4/15 (26.67%) (3)	3/15 (20%) (3)	
Arm C	9/15 (60%) (3)	11/15 (73.33%) (3)	12/15 (80%) (2)	0.47
	6/15 (40%) (3 in four patients and 4 in two patients)	4/15 (26.67%) (3 in two patients and 4 in two patients)	3/15 (20%) (3 in two patients and 4 in one patient)	
P-value	1	0.89	0.87	

**Table 11:** Decrease in analgesic requirements based on the WHO step ladder for cancer pain management. Arm A = 8 Gy in a single fraction; Arm B = 20 Gy in five fractions (4 Gy/fraction); Arm C = 30 Gy in 10 fractions (3 Gy/fraction). WHO: World Health Organization

Variable	At one week (Response in percentage) (WHO step)	At one month (Response in percentage) (WHO step)	At three months (Response in percentage) (WHO step)	P-value
Arm A	09/15 (60%) (2)	10/15 (66.67%) (2)	11/15 (73.33%) (2)	0.74
	6/15 (40%) (3)	5/15 (33.33%) (3)	4/15 (26.67%) (3)	
Arm B	9/15 (60%) (2)	11/15 (73.33%) (2)	12/15 (80%) (2)	0.47
	6/15 (40%) (3)	4/15 (26.67%) (3)	3/15 (20%) (3)	
Arm C	9/15 (60%) (2)	11/15 (73.33%) (1–2)	12/15 (80%) (1–2)	0.47
	6/15 (40%) (3)	4/15 (26.67%) (3)	3/15 (20%) (3)	
P-value	1	0.89	0.87	

## Discussion

Dealing with symptomatic bony metastases can present a challenge in palliative care. While several treatment options are available, external beam radiotherapy remains the primary treatment for painful bone metastases. In our study, we found that all three treatment groups were equally effective in reducing pain, improving performance status, and decreasing the need for pain medication during a three-month follow-up period. The initial pain relief observed with both single-fraction and multifraction radiotherapy indicates that the pain relief mechanism is not related to the reduction of tumor size. Instead, it is more likely due to the local micro-environment changes, leading to the activation of bone resorption by osteoclasts. Hence, there is a lack of dose-response relationship [15]. This hypothesis of pain relief helps explain the higher rates of retreatment after a single dose of 8 Gy treatment because fewer cells will be killed in comparison to 30 Gy in 10 fractions, as reported by the landmark trials on palliative radiotherapy for bone metastases [11,15-18].

According to this study, both single-fraction and multifraction radiotherapy provided pain relief and overall response to 80%-90% of patients observed over three months. The difference between the two methods was not statistically significant. These findings are consistent with previous trials on palliative radiotherapy for bone metastases [11,15-18]. For patients with advanced disease and a short life expectancy and who have a poor ECOG score, the primary goal should be pain relief. In such cases, it is recommended that 8 Gy single fractions be used for palliation of painful bone metastases. This option is preferred due to its greater convenience, lower cost, and shorter duration of hospital stay while maintaining the same efficacy. Analgesic use is also changed on the WHO step ladder scale. Our study showed a change in analgesic use from WHO step ladder 3 to 2 in 20% of patients and the same percentage from WHO step ladder 2 to 1. This suggests that even a single high dose of palliative radiation therapy has a comparable outcome compared to prolonged multifraction palliative radiation therapy.

A previous study evaluated the effectiveness of a single 8 Gy fraction as a treatment option for uncomplicated symptomatic bone metastases and patients with poor performance scores. The study’s findings were comparable with those of a prospective study that assessed pain relief, performance score, and analgesic use [19]. The study concluded that an 8 Gy single fraction could be a viable treatment option for patients with uncomplicated symptomatic bone metastases and poor performance scores. No significant differences were found among the three arms regarding the outlined objectives. However, the single-fraction arm may be preferred for non-ambulatory patients due to fewer visits required. Other factors, such as treatment efficacy, patient comfort, and cost-effectiveness, should also be considered. Further broad and longer investigations are needed to determine the optimal treatment approach for this patient population.

Limitations

The observations require further validation through more extensive sample-sized studies. Notably, our study had some limitations, mainly due to its retrospective design. To obtain more comprehensive and reliable data, a prospective randomized study would be necessary. Another limitation is that some patients were undergoing palliative chemotherapy, hormone therapy, and bisphosphonate treatment during palliative radiation therapy, which we did not analyze separately. Extended follow-up is necessary for a more comprehensive analysis of surviving patients.

## Conclusions

The determination of an appropriate radiation therapy regimen for cancer patients is a multifaceted process that is contingent upon several factors, including the overall health condition and treatment objectives of the patient. For patients with poor performance scores, the preferred approach is the administration of a single fraction of palliative radiation therapy. Conversely, for patients with a life expectancy exceeding six months and a primary treatment objective of pain relief, as well as secondary goals such as tumor debulking and prevention of pathological fractures, a larger dose of multifractionated radiation therapy is the recommended course of treatment.

It is important to note that this study is retrospective and based on a limited sample size. Thus, a more comprehensive investigation is required to obtain more conclusive and consolidated results. Despite the small sample size and limitations, our study has yielded results that can assist fellow clinicians in making informed decisions. Further research is advised to ensure that the appropriate radiation therapy regimen is determined for cancer patients to achieve the best possible outcomes.
